# Implementing non-invasive prenatal testing into publicly funded antenatal screening services for Down syndrome and other conditions in Aotearoa New Zealand

**DOI:** 10.1186/s12884-017-1535-x

**Published:** 2017-10-04

**Authors:** Sara Filoche, Fiona Cram, Bev Lawton, Angela Beard, Peter Stone

**Affiliations:** 1 Women’s Health Research Centre, Department of Obstetrics and Gynaecology, University of Otago, Wellington, New Zealand; 2 Katoa Ltd, Auckland, New Zealand; 3 Christchurch Obstetric Associates, Christchurch, New Zealand; 4 Department of Obstetrics and Gynaecology, Faculty of Medicine and Health Sciences, The University of Auckland, Auckland, New Zealand

**Keywords:** NIPT, Cultural responsiveness, Equity, Implementation, Health service, Antenatal screening

## Abstract

**Background:**

Non-invasive prenatal testing (NIPT) is a relatively new screen for congenital conditions – specifically, common fetal aneuploidies including Down Syndrome. The test is based on isolating freely circulating fragments of fetal-placental DNA that is present in the mother’s blood. NIPT has a superior clinical performance compared to current screening, and has been available privately in Aotearoa New Zealand for the last 4 years.

**Main issue:**

The proposed implementation of NIPT as a publicly funded service may widen the inequity in access to optional antenatal screening that already exists in this country.

**Conclusion:**

This paper discusses precautions that can be taken at the health system, organisation, and personnel levels to ensure that access to NIPT is equitable, that services are culturally responsive, and women’s informed choice is promoted and protected. The adoption of NIPT into publicly funded services is an example of how genetic screening is becoming mainstreamed into health services; as such our approach may also have relevance around the introduction of other genetic and genomic screening initiatives.

## Background

Non-invasive prenatal testing (NIPT) is a new advanced screen for congenital conditions – specifically, common fetal aneuploidies including Down Syndrome. It has been available in Aotearoa New Zealand for the last 4 years and has a superior clinical performance compared to current screening [1–4]. Although NIPT is not presently publicly funded in New Zealand, this is likely to change soon. This paper discusses the clinical performance of, and equity of access to current and NIPT screening options. The potential implementation of NIPT as a publicly funded service is then canvassed, and steps recommended for mitigating against current and emerging inequities.

## Antenatal screening for down syndrome and other conditions

Antenatal screening for Down syndrome and other conditions is often a polarizing issue– with many social, cultural and ethical factors playing out around access, the offer of the screen and the decision by the woman to have it. For some women having the screen is an integral part of their pregnancy journey, and informs the pregnancy outcome. For other women, the pregnancy outcome does not matter, and the additional knowledge about the fetus is irrelevant to whether the pregnancy will continue. The view of the pregnant woman may very well sit in contrast to a medical one, where in addition to the offer to find out about the health of the baby, it may be perceived that the focus is on treating or preventing an infant born with a congenital condition that may have considerable health issues.

## Clinical performance of current screening and NIPT in Aotearoa New Zealand

Antenatal screening for congenital conditions has been available to pregnant women in Aotearoa New Zealand since 1968 and health practitioners “have an obligation under the Primary Maternity Services Notice 2007, issued pursuant to section 88 of the New Zealand Public Health and Disability Act 2000, to advise women of screening services available that are endorsed by the Ministry of Health, including antenatal screening for Down syndrome and other conditions” [5]. Current first trimester screening for conditions such as Down Syndrome, Edward Syndrome and Patau Syndrome comprises the combined results of a blood test and a nuchal translucency ultrasound scan with other information (e.g. maternal age) to give a risk result. There are significant concerns about the quality of screening in New Zealand [6]. Unsafe practices were identified in 2005 including the use of outdated testing, inequity of access, lack of knowledge amongst health care professionals, no process to audit practice, and a lack of adequate information and counselling for parents [6]. The clinical performance of current screening is also low – there is only a 5% chance that the screen correctly detects a baby with Down syndrome [7], and the mother will likely undergo unnecessary invasive diagnostic testing, for which there are is an associated risk of miscarriage – reported as 0.1% [8] to 1% [9].

NIPT is a screening test based on a maternal blood sample and isolating freely circulating fragments of fetal-placental DNA [1–4]. This DNA is then analysed for abnormalities of specific chromosomes associated with conditions such as Down syndrome. NIPT samples are sent overseas for analysis, with the health information stored by the company. For example, Wellington SCL (community laboratory) offer Harmony™ NIPT (Ariosa). The blood sample is processed locally and sent to the USA for analysis (where of note, women can opt in or out of consenting to anonymised laboratory development and validation studies).

NIPT has a very high sensitivity and specificity, and is reported as 99% sensitive for Down syndrome, and up to a 90% likelihood of correctly identifying a pregnancy with Down Syndrome [10]. As with current screening, NIPT does not cover all possible conditions – and a negative screen for Down syndrome does not mean that child will not be born with another condition.

## Access to current screening in Aotearoa New Zealand

Undergoing screening is optional, and access to this service is influenced by a number of factors [5, 11–14], from not being offered the screen in the first instance [15] to barriers (e.g. financial hardship) to accessing screening (e.g. ultrasound) [5, 16]. Current evidence indicates that access (both offer and uptake) is not equitable in the current system [17]. There is differential access to screening by ethnicity, where Māori and Pacific women have low screening rates compared to New Zealand European and Asian women – with anecdotal reports of Māori and Pacific women not being offered screening based on practitioner beliefs that “*they* don’t want it.”

The highest rate of completed screens is reported as 66.5% of births in 2012 to 2013 (the most up-to-date information available at the time of submission) [17]. It is possible that this figure reflects the demand for an antenatal screening service, or it may be that with a more robust screen (such as NIPT) that was widely available and offered in a culturally responsive way, more women would opt to undergo screening. There has been limited public discussion around antenatal screening for congenital conditions, and given that NIPT may be adopted into screening services for Down syndrome and other conditions it may be timely to discuss the future of this service.

## Access to NIPT in Aotearoa New Zealand

Currently NIPT is available to all women in New Zealand through a user pays service (a for-profit-model). Even within a user pays model, anecdotal evidence suggests that NIPT is being accessed on an adhoc basis, with only some district health boards (DHBs) offering an appointment for the blood draw; and if this is not offered by a DHB, a woman will need to pay for a private consultation in addition to the NIPT screen. The cost of an NIPT screen is around $600, rising to over $2000 with expanded screens. Access to NIPT is therefore based on women (most likely through their health providers) knowing about this new screening technology and then opting to pay for it. There is currently no published information on the numbers of women who are opting for NIPT in Aotearoa New Zealand. However, given the cost of the service and the socioeconomic disparities that exist for Māori and Pacific women in this country, it is highly plausible that Māori and Pacific women are not accessing NIPT even in the absence of cultural concerns about this service.

## Implementation of NIPT into a publicly funded service

The National Screening Unit (NSU) and Ministry of Health are currently exploring the implementation of NIPT in Aotearoa New Zealand, and it will likely be funded in some form. This may be as a contingent screen based on high risk from current screening, with a limited number of conditions being screened for, as implemented in the UK [18], and Canada [19]. Emerging evidence, however, indicates that women want NIPT as a first line option [19], as do maternity care providers [20].

**Fig. 1:**
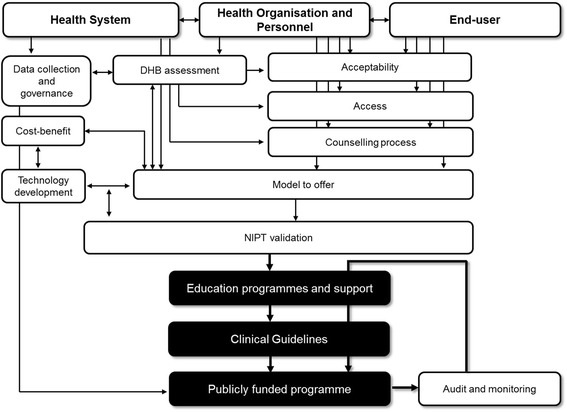
Capacity building at health system, organisation and personnel level with end-user input and suggested steps (areas of work) to the implementation of NIPT as a publicly funded service, building on Jansen, Bacal and Crengle [21] and Cram [22]

The adoption of NIPT into publicly funded services is an example of how genetic screening is becoming mainstreamed into health services. We are proposing that the successful implementation of NIPT is contingent upon the capacity of the health system to offer a culturally responsive and accessible screening service. Following on from the work of Jansen, Bacal and Crengle [21] and Cram [22] we propose that capacity needs to be built at the health system level, organisation and personnel levels, which may include specific steps outlined in Fig. 1, with input also from end-users (pregnant women). These are explored below after an overview of potential cultural concerns Māori women and their families may have about NIPT. We are also proposing that there needs to be a sustained audit and evaluation programme once NIPT has been implemented (Fig. 1).

## Engaging Māori

Given the inequities that exist in the current antenatal screening it is likely that these will perpetuate with the roll out of NIPT unless action is taken to mitigate it. In terms of implementation of NIPT into a public programme, an important first step would be to explore the needs of Māori women and whānau (families) around screening for congenital conditions.

For Māori, everything in the natural world possesses mauri (life force), and everything is inter-related and inter-connected through whakapapa (genealogy) [23, 24]. Smith and Reynolds [25] described the importance of maintaining the sanctity of whakapapa; that no one should interfere or corrupt it. Although their focus was on genetic modification the same respect for whakapapa flows through much of Māori thinking and practice, and is an important consideration for NIPT. When asked directly about pre-birth genetic testing and abortion Māori hold a broad range of views. Even so, the strengthening of whakapapa and ensuring the collective’s wellbeing are central considerations that in the past have led to taumau (arranged marriage), spiritual interventions before and during pregnancy, abortion and infanticide [26]. As Tipene-Matua and Henaghan [26] speculate, “pre-birth genetic testing may be a contemporary extension of such cultural practices.” Indeed, Māori with pre-existing conditions have embraced prenatal testing [27].

## Health personnel

Work in New Zealand is already underway to explore provider viewpoints, with 108 maternity care providers indicating “general enthusiasm” towards the introduction of publicly funded NIPT [28], and call for more practice support by obstetrics professionals [29]. Evidence from overseas suggests that the successful integration of NIPT into antenatal care will be reliant upon the competencies and practice patterns of maternity care providers [7]. In a recent study of Māori access to cancer, cardiovascular and cancer health services key informants identified three inter-related aspects of health practitioner practice that were foundational to the provision of clinically and culturally competent health care (as proscribed by the Health Practitioners Competency Act 2004): establishing relationships, building rapport and communicating, and Māori-centred clinical practice [22]. Māori-centred clinical practice works with whānau and respects their collective decision-making processes [27]. For example, communication of the results of NIPT with Māori might follow the recommendation of Port et al. [27]; namely, that clinicians and whānau learn the results together. This was seen as a way of reducing prejudice as well as a means of promoting collective decision-making about next steps if a test is positive. As Port, et al. [27] note, for Māori “[a]utonomy may be within a collective rather than an individual worldview.”

Health practitioners will also need to build their knowledge about this new screening paradigm. As the number of conditions that NIPT includes increases, it is likely that there will be findings of unknown medical significance. Health practitioners may need to explain to women statements in reports such as “uninformative DNA pattern” (Panorama™, 2013, guide to patients), or even unexpected findings such as maternal malignancy [30]. It is not yet known how local maternity care providers will become “NIPT literate”, and this will be key part in implementation.

## Health organisations

A significant body of work around biobanking and genetic testing exists for Māori; potentially there are lessons that could be learnt from this work for the introduction of NIPT [31–33]. Implementation of NIPT could also follow a model and process that was used to develop a more culturally sensitive genetic service in Auckland DHB – for example establishing a position for a Kai Arataki, a cultural adviser, within the genetic service [27].

Appropriate protocols need to be developed around informed consent and blood draw (and laboratory processing). These protocols are also pivotal to ensure good sharing of information with women and the organisational responsibility to enable health practitioners to increase patients’/women’s health literacy.

It will also be important to assess, and monitor, how to offer NIPT in terms of who provides counselling and how informed consent with NIPT will be reconciled with the reality of (over) busy maternity care providers and clinics. At the same time it will also be important that women have access to bespoke information and counselling. Paramount also, is the infrastructure to enable a woman to make her decision about whether or not to participate in antenatal screening, and this may mean more time and/or a separate visit for counselling.

## Health system

As the Government body that provides screening for Aotearoa New Zealand, the NSU will have overall responsibility for the implementation and management of the introduction of NIPT and with that comes due diligence around for example, ensuring appropriate data collection systems are in place, governance of health information, model of NIPT that is offered and technology development - all of which would need to be worked out with district health boards, and providers to ensure that the right infrastructure (from booking system, follow-up, consultation times to education programmes) is in place (Fig. 1). In terms of information storage (health information from NIPT is currently stored overseas) this should be taken with a long-term view, in light of expanded screens that cover adult onset conditions.

Culturally responsive NIPT practices will need to acknowledge the diversity of views held by Māori, while those in charge of the screening initiative may be well advised to form a Māori kaitiaki (caretaking) group. The role of such a group would be to maintain the sanctity of whakapapa in these modern times when access to cultural knowledge and resources is not guaranteed for all Māori women and whānau. This includes addressing Māori concerns about their DNA being sent offshore for analysis [27]. The formation of a rōpū kaitiaki (caretaking group) would be an appropriate partnership response under Article 2 of the Treaty of Waitangi (signed in 1840 between Māori chiefs and the British Crown), recognizing that Māori genes and DNA are taonga (treasures) [27].

An offer of a publicly funded contingent NIPT screening may not be acceptable to some women. Most companies have expanded their NIPT screen to include microdeletions and other conditions as part of their ‘standard’ screen. Furthermore, NIPT is advertised commercially, with dialogues describing how a woman and her family would “pay money, over and over again for the security that they felt from having the test” (BGI Diagnostics, 2014). The consumerism aspect of NIPT may place additional pressure, with an accompanying financial burden for privately funded screening, on women who feel that they should have this new ‘standard’ of an expanded and more expensive screen. This, in turn, could reduce the number of women accessing a publically funded service and impact on the services’ utility. The offer of a publicly funded limited screen may therefore be regarded as the ‘poor man’s choice,’ prompting inequity of access from the outset between those who pay and those who do not.

## Recommendations for implementation

We are proposing several recommendations for the implementation of NIPT that would need to be addressed at health system, health organisation, and health personnel levels to ensure that access to NIPT is equitable, that services are culturally responsive, and women’s informed choice is promoted and protected. Specifically, we recommend:

- input from end-users, in particular from Māori, in terms of developing a culturally appropriate informed decision making process for NIPT.
- capacity assessment at DHB levels for example in terms of pre-and post screen counselling, and sample processing.
- clinical validation of NIPT based on the model adopted in this country that has been developed inline with provider and end-user input, which may include the development of NIPT technologies in Aotearoa.
- development of appropriate governance of genetic health information that could potentially be gained from a public screening service.

While our paper focusses on the implementation of publicly funded NIPT in New Zealand, we propose that the principles and recommendations for engaging groups that traditionally have been disenfranchised, or where barriers in access to healthcare services exist, would be applicable internationally. The adoption of NIPT into publicly funded services is an example of how genetic screening is becoming mainstreamed into health services; as such our model may also have relevance around the introduction of other genetic and genomic screening initiatives.

## Conclusions

New Zealand appears to have come to a cross-road around antenatal screening for congenital conditions. The arrival of NIPT promises a real opportunity to improve screening for congenital conditions. We have the evidence to learn from what is not working currently [6, 15, 16] and turning this into something that does; implementing NIPT as a publicly funded service will require a multilayered approach – taking into account the needs of the women using the service and those that provide it, and for these to be incorporated into the system that will administer it. To not seize this opportunity would do New Zealand women a great disservice.

## Abbreviations

DHB: District Health Board
DNA: Deoxyribonucleic acid
NIPT: Non-invasive prenatal testing
NSU: National Screening Unit
